# Food Quality Affects Secondary Consumers Even at Low Quantities: An Experimental Test with Larval European Lobster

**DOI:** 10.1371/journal.pone.0033550

**Published:** 2012-03-19

**Authors:** Katherina L. Schoo, Nicole Aberle, Arne M. Malzahn, Maarten Boersma

**Affiliations:** 1 Alfred-Wegener-Institute for Polar and Marine Biology, Biologische Anstalt Helgoland, Helgoland, Germany; 2 GKSS Research Centre, Institute for Coastal Research, Geesthacht, Germany; Institute of Marine Research, Norway

## Abstract

The issues of food quality and food quantity are crucial for trophic interactions. Although most research has focussed on the primary producer – herbivore link, recent studies have shown that quality effects at the bottom of the food web propagate to higher trophic levels. Negative effects of poor food quality have almost exclusively been demonstrated at higher food quantities. Whether these negative effects have the same impact at low food availability in situations where the majority if not all of the resources are channelled into routine metabolism, is under debate. In this study a tri-trophic food chain was designed, consisting of the algae *Rhodomonas salina*, the copepod *Acartia tonsa* and freshly hatched larvae of the European lobster *Homarus gammarus*. The lobster larvae were presented with food of two different qualities (C∶P ratios) and four different quantities to investigate the combined effects of food quality and quantity. Our results show that the quality of food has an impact on the condition of lobster larvae even at very low food quantities. Food with a lower C∶P content resulted in higher condition of the lobster larvae regardless of the quantity of food. These interacting effects of food quality and food quantity can have far reaching consequences for ecosystem productivity.

## Introduction

The quality and quantity of food available to consumers influences development, growth and reproduction and thereby shapes trophic interactions and food web dynamics. In ecological stoichiometry food quality is defined by the ratio of carbon to nutrient content of the food [1], [2]. In the aquatic environment, herbivorous consumers are often faced with low quality food as plants contain high amounts of C and relatively low amounts of P and N, making the latter nutrients potentially limiting [1], [3]. Furthermore, the nutrient content of aquatic primary producers undergoes changes and reflects that of its surrounding environment [4]. Herbivores therefore regularly face imbalanced food in terms of energy and nutrients, which negatively affects their growth, development and reproduction [1]. Under low quality food conditions (high C:nutrient content) animals are faced with an excess of carbon in their food. Among the mechanisms herbivores have developed to cope with this surplus of carbon are increased respiration [5], potentially through an increase in activity [6] or excretion [7]. Whichever mechanism is employed, it comes at a cost to the animal, usually resulting in reduced condition and fitness. Furthermore, under low nutrient conditions growth and reproduction can be nutrient limited, as both N and P are required for production [8], [9], [4]. Low algal food quality will result in reduced population growth rates of herbivores, resulting in a decrease of ecosystem productivity.

It has been hypothesized that the effects of nutrient limitation are important at higher quantities of food only, because at low quantities the production of new tissue is low, and hence the requirement for structural building blocks containing phosphorus or nitrogen is also low [11], [12]. Basic routine metabolism dominates and this metabolism requires mostly carbon. If the amount of available food is too low to provide the organism with sufficient energy for growth, the metabolic energy requirements for maintenance must be met first, theoretically, as no new tissue is synthesised, only requiring carbon. Therefore, at low quantities, food quality effects should not be present [11]. In contrast, at higher food quantities food quality should play an important role, as growth would no longer be C-limited and mineral nutrients would be needed for production [11], [13].

The studies conducted on the interaction of food quality and food quantity have so far concentrated on the primary producer-herbivore interface [14]. One of the main reasons for this focus is the stoichiometry theory, which assumes that the consumers maintain homeostasis to a large extent, i.e. a constant ratio of carbon to nutrients [1], [15]. Negative effects of low quality are therefore thought to be buffered by the primary consumers and should not affect higher trophic levels. Recent studies have shown, however, that the homeostasis in herbivores is far from perfect and that the effect of low quality food can be traced to higher trophic levels via the herbivores [16], [17], [18], [19], [20], [21]. Hence, the question of how higher trophic levels react to food of simultaneously low quality and low quantity requires further study and is relevant to our understanding of trophic interactions and food web dynamics.

Here, we investigate the effect of different qualities (C∶P ratios) and quantities of food on the condition of larvae of the European lobster, *Homarus gammarus*. The lobster is an important top predator around Helgoland in the North Sea, and plays a crucial ecological role in maintaining the species diversity of the local community [22]. The population numbers have been in steady decline since the 1940s, making this lobster community particularly vulnerable. By constructing a laboratory based three-trophic food chain in which the quality of the food was manipulated separately from the quantity we aim to disentangle the potentially confounding effects of these two factors.

## Materials and Methods

In order to investigate the effect of low and high quality food on a secondary consumer, a three-trophic food chain was established, consisting of algae (*Rhodomonas salina*), copepods (*Acartia tonsa*) and lobster larvae. The algae and the copepods were reared under nutrient-replete and phosphorus-limited conditions in order to manipulate their nutrient ratio and hence their quality. Copepods of the two resulting different qualities (C∶P ratios) were then fed to the lobster larvae at different concentrations, simultaneously exposing the larvae to differences in the quality and the quantity of their food (for more detail on analytical procedures see also [16]).

### Phytoplankton

A stock culture of *R. salina* was cultivated in enriched seawater (f/2) according to Guillard and Ryther [23]. For the experiment, all algal treatments were grown under a 16∶8 h light∶ dark (L∶D) regime at a constant temperature of 18°C. The seawater used for the growth media in the experiments was filtered using a sterile 0.2 µm filter and stored cool and dark until use. The seawater for the nutrient-replete f/2 treatment was enriched with the full set of nutrients as described by Guillard and Ryther [23], and used as algal culture medium for the f/2 treatment. The water for the growth medium of the P-limited treatment was also enriched according to the f/2 recipe, but without the addition of the limiting nutrient P (-P treatment). The algae thus had access only to the P contained in the natural seawater at the time of filtration and previous tests of the carrying capacity showed that the algae became severely limited with respect to the missing nutrient after a growth period of 4 days.

Algal concentrations in the stock solution were measured with a CASY cell counter (Schärfe System CASY Cell Counter and Analyser System). To ensure a constant supply and food quality, new cultures of *R. salina* were inoculated daily for both treatments with a starting concentration of 0.2×10^6^ cells L^−1^ for the f/2 treatment and 0.3×10^6^ cells L^−1^ for the –P treatment. Algae were harvested after the predetermined growth period of 4 days at concentrations of 1.5×10^6^ cells L^−1^ for the f/2 treatment and 1.0×10^6^ cells L^−1^ for the –P treatment. For the feeding experiment the amount of algal food fed to the copepods was normalized to the same concentrations for both food qualities. .

### Zooplankton

Copepod eggs were obtained from a culture of the calanoid copepod *A. tonsa*. The copepods for egg production were kept in filtered natural seawater (salinity ∼32) in a 200 L tank at 18°C under a 18∶6 L∶D regime. The copepods were fed a mixture of the algae *R. salina* and the heterotrophic flagellate *Oxyrrhis* sp. Eggs were siphoned off the bottom of the tank daily and stored in seawater under anaerobic conditions in the dark at 4°C until use. No eggs older than 3 months were used in the experiments.

The copepod eggs were incubated at a concentration of about 3000 individuals L^−1^ in artificial seawater (salt: hw Marinemix, www.hw-wiegandt.de) adjusted to a salinity of 32. Hatching occurred within 24 hours. Hatching rate was approximately 20%. Copepods were first fed 48 hours after the addition of the eggs to seawater, when the majority had reached the second naupliar stage, which is the first feeding stage in *A. tonsa* nauplii [24]. The copepods were transferred to fresh artificial seawater daily prior to feeding in order to avoid nutrient uptake of the nutrient-limited algae from waste products of the copepods. The copepods were fed 50,000 cells of *R. salina* per individual and day (corresponding to ∼3 mg C L^−1^ d^−1^), irrespective of the nutrient treatment. This concentration is considered *ad libitum* for juvenile copepods. Copepods were fed the different algae treatments for a total duration of 8 days, after which period of time the majority of the copepods had reached the third copepodite stage. Previous tests had shown that this copepodite stage represents a good prey size for the lobster larvae. To ensure a constant food quality for the secondary consumers for every day of the feeding experiment new copepod cultures were started for each of the nutrient treatments. Since the copepods fed on P-limited algae displayed slower growth and development, the copepod cultures fed f/2 algae were started one day later. This delay was necessary to ensure that all the copepods fed to the secondary consumers were of the same age and size class. Copepods were decanted over a sieve and transferred to fresh artificial seawater prior to being fed to the lobster larvae to ensure the lobsters were fed only the copepods.

### Lobster

Freshly hatched stage 1 larvae of the European lobster *Homarus gammarus* were collected from the lobster rearing facility at the Biological Station on Helgoland, Alfred-Wegener-Institute for Polar- and Marine Research (AWI). We used young larvae, as they have been shown to be particularly sensitive to differences in feeding environment [25]. Larvae from different females were collected just after hatching to minimize batch difference and possible maternal effects. The larvae were carefully sorted and each larva was transferred to an individual 40 mL cylindrical glass container filled with sterile filtered natural seawater. The water in the beakers was changed daily prior to feeding. The lobsters were kept at a 16∶8 L∶D regime under indirect light in a temperature-controlled room at 15°C, resulting in a time frame of approximately five days until first moulting [26], [27].

Ten lobster larvae were randomly assigned to each of the quality and quantity treatments. Each larva was kept in a separate container and reared individually. Copepods were fed to the lobster larvae along a quantity gradient. There were four quantity treatments for each of the two qualities (f/2 and –P). The highest food concentration consisted of 60 copepodites per lobster larva and day. This amount was *ad libitum* in preliminary tests (Schoo, unpublished data).To test the effect of different food quantity, the food concentration was reduced stepwise to result in 4 different food quantity treatments (60, 30, 15 and 7 copepodites per lobster larva and day, expressed as µg C) per food quality (-P and f/2 copepods, respectively). An additional 10 lobster larvae were assigned to a starvation group. First feeding took place within 12 hours of hatching. The larvae were provided with food once a day.

On the fifth day after hatching the lobster larvae were sampled, rinsed in distilled water and frozen at −80°C until analysis.

### Analytical procedures

The stoichiometry of each of the three levels of the food chain was analyzed. For the analysis of the primary producer *R. salina* approximately 4×10^6^ cells were filtered onto pre-combusted and washed Whatman GF/F filters. For each of the analyses of the copepods 75 individuals were counted into tin capsules. To assess the nutritional condition (referred to as condition in this study) of the larvae of *H. gammarus* fed prey of differing nutritional value, we used the RNA∶DNA ratio of the lobster larvae.. For the analysis of the RNA∶ DNA ratio of the larvae, whole freeze dried *H. gammarus* larvae were used. The technique to measure RNA and DNA content of animal tissue is well established and commonly used especially in fisheries science to determine the overall physiological condition and growth (e.g. [28], [29], [30]). A high RNA to DNA ratio is indicative of a good overall nutritional condition, as the DNA content of the cell should be constant relative to RNA, which should increase under active growth conditions. Lately the technique has also been successfully applied to determine the condition of crustaceans (e.g. [31], [32]), and hence the RNA∶DNA ratio will be used as the measure of the nutritional condition of the lobster larvae in this study.

The carbon and nitrogen content of the samples was measured with an Elementar vario MICRO cube CHN analyser (Elementar Analysensysteme, www.elementar.de).

Phosphorus was analysed as orthophosphate, after the method described by Grasshoff et al. [33], following oxidative hydrolysis. The samples were treated with an oxidation agent (K_2_S_2_O_8_, H_3_BO_3_, NaOH in distilled water) under high pressure and at high temperature (120°C) in an autoclave to convert the phosphorus compounds to the ortho-phosphate form. Molybdate-antimony-solution (containing ammonium molybdate (NH_4_)_6_Mo_7_O_24_×4H_2_O, antimony potassium tartrate K(SbO)C_4_H_4_O_6_×0,5H_2_O) and ascorbic acid was added to the solute before the P-content was measured photometrically.

The method used for the analysis of nucleic acids was modified from Clemmesen et al. [29] as described in Malzahn et al. [16]. For the analysis of the RNA∶DNA ratio entire freeze-dried larvae were pulverised and rehydrated in 400 µl Tris-SDS buffer (Tris 0.05 mol L^−1^, NaCl 0.01 mol L^−1^, EDTA 0.01 mol L^−1^, sodium dodecyl sulphate (SDS) 0.01%) for 30 minutes. Glass beads (2 mm and 0.17–0.34 mm diameter) were added and the tubes containing the samples shaken in a Retsch MM 301 cell-mill for 15 min. The homogenate was centrifuged (Sartorius Sigma 3–18 K; 8 min, 3800 g, 4°C) and 130 µl of the supernatant used for analysis. The amount of nucleic acids was determined fluorometrically using the fluorophor ethidiumbromide (EB) in a microtiter fluorescence reader (Fluoroskan Ascent). The total amount of nucleic acids in the sample was measured first, before the addition of RNAse to digest the RNA. The remaining DNA was measured after the enzyme treatment and the RNA fluorescence calculated by subtracting the DNA fluorescence from the total nucleic acid fluorescence. The DNA concentrations were calculated following Le Pecq and Paoletti [34]. RNA calibrations were set up daily prior to the measurements.

### Statistical analysis

The C∶P ratios of the primary producer *R. salina* and the copepod *A. tonsa* were analysed with a one-way ANOVA (StatSoft Statistica 7), using the C∶P ratio as the dependent variable and the nutrient treatment (f/2 and –P, respectively) as the independent variable.

A linear regression analysis was performed to test the effect of quality (C∶P ratio of the food) and quantity (amount of carbon in the diet) on the response of the lobster larvae, expressed by the RNA∶DNA ratio of the larvae. After a test for homogeneity of slopes, an ANCOVA was carried out to test for the effects of both quality and quantity of the food on the condition of the lobster, with ‘condition of the lobster larvae’, expressed by the RNA∶DNA ratio, as the dependent, ‘Quality’ as the categorical and ‘Quantity’ as the co-variable.

Tukey's honest significant difference test was used as the post hoc test in all cases.

## Results

**Figure 1:**
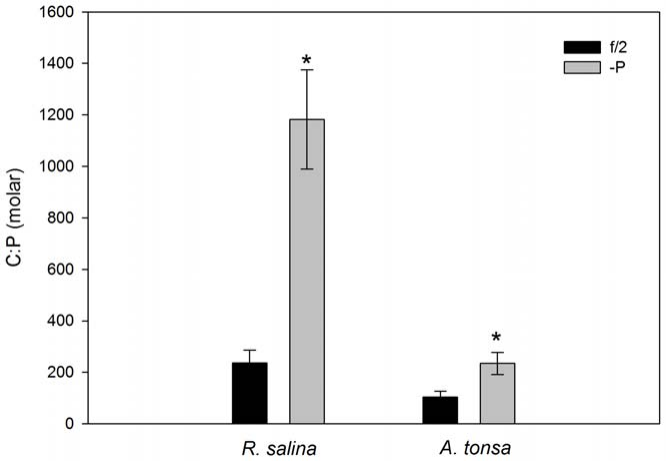
Food quality (C∶P ratio) of the primary producer *R. salina* and the primary consumer *A tonsa*. Stars mark significant differences between treatments.

The differently manipulated growth media resulted in two algae cultures of very different quality. P-limited algal cultures yielded much lower cell concentrations than the nutrient-replete algae. The C∶P ratios of the algae grown in the nutrient-replete f/2 growth medium were significantly different from those of the –P algae (Figure 1; f/2 ∼230, -P ∼1180; ANOVA: p<0.001).

The same pattern was visible in the primary consumer, where the copepods fed the f/2 diet had a mean C∶P ratio of ∼100, while the C∶P ratio of –P copepods was elevated at ∼230 (Figure 1). These differences were statistically significant at p<0.005. Thus the low quality (high C∶P) of the algae was still visible in the copepods, which were unable to maintain complete homeostasis.

Copepods reared on P-limited algae contained 0.36 µg C on average per individual copepod, compared with an average of 0.5 µg C in each copepod reared on f/2 algae. The carbon content of the copepod prey differed slightly between the two treatments (copepods reared on P-limited algae contained less C than those reared on f/2 algae), resulting in a lower amount of food for the lobster larvae in the –P treatments throughout. The copepods had reached the same developmental stages regardless of the food quality they were reared on and the results were analysed with linear regression analysis, where food quantity available to the lobster larvae was expressed as the average amount of carbon contained in the copepodites from the different treatments.

Significant differences in condition (RNA∶DNA ratios) were observed between lobsters from the starvation treatment and the lowest P-limited food quantities (ANOVA, F_(8,54)_ = 13.26, p<0.01), as well as between the starved larvae and the highest f/2 food quantities (ANOVA, F_(8,54)_ = 13.26, p<0.01).

**Figure 2:**
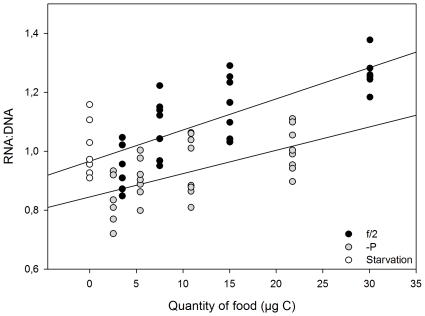
RNA∶DNA ratio of the lobster larvae and food quantity expressed as the amount of carbon in the diet.

The RNA∶DNA values of the lobster larvae were positively related to food quantity (expressed as C in the food, Figure 2) for both high (linear regression: y = 0.0106x+0.966, r^2^ = 0.58; p<0.001) and low (linear regression: y = 0.0079x+0.8447, r^2^ = 0.34; p<0.05) food qualities. No statistically significant interaction was detected between the quality and the quantity of the food (linear regression analysis, F _(1,52)_ = 0.877; p = 0.35) and thus the assumption of homogeneity of the regression slopes was met. An Analysis of Covariance (ANCOVA) was run showing statistically highly significant effects for food quantity (p<0.001) and quality (p<0.001). These results indicate that the condition (RNA∶DNA ratio) of the lobster larvae is affected by both the quantity and the quality of their food. The reactions of the lobster larvae to the different qualities of food (as shown by the regression slopes) were not significantly non-parallel (the slopes did not differ), showing that the food quality affected the larvae irrespective of the food quantities. This is true even for very low food quantity.

Differences in the behaviour of the fed and non-fed larvae were also observed, with the fed animals displaying sustained bursts of swimming activity during their hunt for the prey items, while the starved animals remained inert and did not show a great deal of activity.

## Discussion

This is the first study on the combined effect of food quality and food quantity on a secondary consumer that we are aware of, building on the previous work of Boersma and Kreutzer [13]. The effect of low quality food, in terms of C:nutrient ratios, on higher trophic levels is under increasing attention and we now know that the nutrient-limitation in primary consumers can be traced to secondary consumers [16], [17], [20], [21].

The quality of the food played a physiologically and statistically significant role at all levels of food quantity. Considering the effect of food quantity on the larvae, the lowest larvae condition was observed not in the starvation group, which received no food at any point, but in the animals having received the lowest food quantities in the experiment. This difference between the feeding and the non-feeding animals can be explained by the metabolic cost of feeding. The metabolic rate of fasting animals is much lower compared to that of feeding animals, as is the rate of respiration under fasting conditions [35], [36], [37]. As soon as the animal feeds, however, the respiration rate increases. This increase above the basic metabolic rate associated with feeding is known as the specific dynamic action (SDA) [35], [37], [38]. SDA reflects the energy requirements resulting from feeding, including physical and biochemical processes such as the capture and handling of prey items, digestion, absorption of nutrients and the deamination of amino acids (for a review see [39]). SDA is a typical metabolic reaction associated with feeding (reviewed by [40]) and has been observed in a variety of crustaceans [2], [39], [41], [42]. Experiments with juvenile rock lobster *Jasus edwardsii*, for example, showed a significant increase in the oxygen consumption rate after feeding, while the oxygen consumption rate of starving larvae remained low [2]. The effect of the SDA in this case lasted for 32 hours, during which time the oxygen consumption rate of the fed lobster remained significantly elevated compared to that of the unfed animals. Such an elevation of energy expenditure can have negative effects on the fitness of the animal, if the energy costs incurred are higher than the energy gained from feeding.

In the present experiment it appears that the metabolic costs associated with feeding at low quantities of food were actually higher than the energy acquired by feeding. The lower food quantities in our experiment apparently did not provide the lobster larvae with enough energy to compensate for the energy expenditure of feeding and accumulation and hence resulted in lower condition (lower RNA∶DNA ratio) of these larvae. With regard to the increasing metabolic activity associated with feeding, the lobster larvae also showed elevated levels of activity during feeding, further increasing their energy demand. A similar pattern has been described for juvenile rock lobster *Jasus edwardsii* [2], as well as for adults of the species [41]. When compared to feeding animals, the non-fed larvae generally displayed lower activity, displaying hardly any movement at all and thus further reducing their energy expenditure similar to an energy-saving modus.

Even though there was not enough energy to be gained for the lobster larvae at the lowest of our experimental food concentrations, the quality of the food ingested had a strong effect. The P-deficient diet resulted in lower condition (RNA∶DNA ratio) of the animals even at the lowest food concentrations. In this respect our results are in line with observations by Boersma and Kreutzer [13] and Kilham et al. [43], who discovered negative effects of low quality food for *Daphnia* even at low C quantities. In their study the authors found quality-dependent shifts in the threshold food concentration required for growth in *Daphnia*, with higher amounts of low quality food required for growth. This is in contrast to the predictions by Sterner and Robinson [12] who postulate that only energy (carbon) is needed to maintain basic metabolism, whereas minerals are needed in addition to carbon only during growth. The mineral limitation in our study, however, had an effect on the lobsters' condition at all food quantities. The lobster larvae in this experiment were freshly hatched and probably had high P requirements to support their fast growth, a pattern which has also been observed in other larvae [1]. The high growth rate in larvae and juveniles requires large amounts of P-rich RNA for protein translation and synthesis [44], making young and rapidly growing animals particularly vulnerable to nutrient limitations. The requirements of juvenile stages with their high growth rate probably differ from those of adult animals, which are likely to invest their resources into maintenance and reproduction [9], [45], [46]. Therefore the demands and responses of the animals and the repercussions to the population will probably vary depending on the ontogenetic development of the animal.

While our food chain represents a laboratory based experiment under artificial and highly controlled conditions, it is certainly relevant to the natural conditions prevailing in the aquatic environment. In previous experiments we have observed lower abundances of primary producers and herbivorous consumers grown under low-quality food conditions, as well as a slower development in copepods (see also [16]). Under experimental conditions we were able to adjust for these differences. In the field, further factors, such as a heterogeneous composition of the prey in terms of age and developmental stage, would come into play, further complicating the issue.

A co-limitation in food quality and food quantity is likely to occur occasionally, even for higher trophic levels, since homeostasis in herbivorous consumers is not as strict as previously assumed. Since not only the fitness, survival and reproductive output of herbivores, but also their biochemical composition, and hence quality as prey for higher trophic levels is determined by the quality of the algal food available to them, food quality and food quantity for higher order consumers are interrelated and affected at the same time. In the field, however, a wide range in diets, including many different prey items or a selective feeding strategy may lessen the impact of quality and quantity limitations on predators by providing a more balanced diet. Our results show that there is, however, a great need for further studies on the combined effects of food quality and quantity on higher trophic levels.
